# jurisprudential and ethical considerations of practicing medical Islamic procedures on nearly dead patients: Part II (Shiite jurisprudents’ viewpoints) 

**Published:** 2018-12-25

**Authors:** Nazafarin Ghasemzadeh, Fariba Asghari, Mandana Shirazi, Fatemeh Faramarzi Razini, Bagher Larijani

**Affiliations:** 1 *PhD Candidate in Medical Ethics, Medical Ethics and History of Medicine Research Center, Tehran University of Medical Sciences, Tehran, Iran. *; 2 *Associate Professor, Medical Ethics and History of Medicine Research Center, Tehran University of Medical Sciences, Tehran, Iran.*; 3 *Associate Professor, Department of Medical Education, School of Medicine, Tehran University of Medical Sciences, Tehran, Iran.*; 4 *Mentor, Department of Jurisprudence and Islamic Law, Urmia University, Urmia, Iran.*; 5 *Professor, Endocrinology and Metabolism Research Center, Endocrinology and Metabolism Clinical Sciences Institute, Tehran, Iran; Medical Ethics and History of Medicine Research Center, Tehran University of Medical Sciences, Tehran, Iran.*

**Keywords:** *Medical training*, *dying patient*, *opinions of contemporary shiite jurisprudents*, *islamic jurisprudential rules*

## Abstract

Part one of the present study presented practical Islamic jurisprudential rules and investigated their application to performing medical procedures on nearly dead patients. It was contended that a dying patient could be used in medical education in cases where there is no alternative method, provided the patient voluntarily consents and is not offended. Part two of the present study addresses the issue by referring to the opinions of Islamic jurisprudents to find an appropriate solution to a challenging question in medicine, namely, whether clinical training of medical students on the dying person is permissible. For this purpose, *istiftas *(petitions or requests for a *fatwa*) were sent to prominent contemporary Shiite jurisprudents to solicit their opinions on the use of dying patients for medical education. After exploring the existing views, it was finally concluded that the majority of the jurisprudents allowed the practice in cases of “necessity” and provided that the principles of “no harm” and “consent” were strictly observed. All these terms are found in jurisprudential rules, and we reached the conclusion that Shiite jurisprudents considered this type of training permissible under certain circumstances and in accordance with jurisprudential rules.

## Introduction

New medical developments give rise to issues to which contemporary Islamic jurisprudents cannot remain indifferent. The reason is that their opinions on upcoming matters are applied as guidelines in different disciplines, medicine included. The importance of jurisprudential rules is asserted in Article 3 of the Code of Civil Procedures of the Islamic of Republic of Iran, which requires judges to base their decisions on jurisprudence when statutes or codes provide no clear answer to the case in dispute (1). Thus, it is important for the medical community to interact with jurisprudents and establish ethical policies to achieve the objectives of medical education and the health system while taking human dignity into account. This study presents the contemporary Shiite jurisprudents’ opinions on the use of dying patients for medical education purposes. In this regard, the authors sent *istiftas *to contemporary Shiite jurisprudents and analyzed their views and arguments in order to present a collective viewpoint on the issue.

## Method

The study consists of both a theoretical (part 1 which was published) and a field (part 2) section. In the field section, a qualitative investigation was conducted by sending *istiftas *(asking for a religious opinion) to prominent contemporary Shiite authorities (*maraje’* *taghlid*). We emailed and posted 6 istiftas (questions on religious matters) to religious Shiite scholars considering the possibility and conditions of practicing and teaching medical procedures on nearly dead patients and the replies were received in a 6-month period. In addition, these questions contained the images of medical procedures for instance, endotracheal intubation, central venous catheter insertion and pericardiocentesis. Religious scholars as sources of emulation who responded to our questions in the present study were Ayatollah Khamenei, Ayatollah Safi Golpayegani, Ayatollah Alavi Gorgani, Ayatollah Fazel Lankarani, Ayatollah Makarem Shirazi, Ayatollah Noori Hamedani, Ayatollah Sistani, Ayatollah Mazaheri and Ayatollah Seyyed Abbas Modaresi Yazdi. Consequently, their viewpoints on performing training activities on nearly dead patients were explained and analyzed. Finally, the results of both sections were summarized and a theory was presented with regard to medical training activities on dying patients.

## Results

*Views of prominent contemporary Islamic jurisprudents*

In order to understand the religious opinions of contemporary Shiite authorities (*maraje’ taghlid*), questions were drafted on using dying patients for medical education (Table 1). The consensus among prominent contemporary jurisprudents indicates that it is often permissible to consider the use of a dying patient under such conditions as “a state of necessity”, “non-harassment” and “permission”. These conditions can be derived from a number of jurisprudential rules. It should be remembered that the discussion of issues such as the rule of authority or the permission and consent expressed by some jurisprudents should not create doubts that autonomy can be reconciled with free will.

## Discussion

As shown in Table 1, the majority of prominent Shiite jurisprudents declared the use of a dying person for medical education permissible under certain conditions, including “necessity”, “no harm” and “consent”. In this regard, they proposed the conventional concept of necessity, which is applicable to particular examples; for instance they believed that medical training on a dying person should be confirmed by conventional medicine. On the other hand, although the jurisprudents recognized the educational value of such activities, they were of the opinion that “necessity must only be assessed proportionately”, which exerts limitations on the whole issue. Thus, medical procedures on dying patients are only acceptable in cases of necessity, and undue implementation or perpetuation of such measures is prohibited. The important question is, what are the criteria and diagnosis authority for the necessity? In other words, who determines the necessity of these activities? It is clear that time and place requirements are involved here, which adds to the sensitivity of the issue.

**Table 1:** The rights of near-death patients regarding using their bodies for medical training purposes according to prominent Shiite scholars based on the obtained *istiftas*

** Answer**	**Ayatollah ** **Khamenei**	**Ayatollah ** **Sistani**	**Ayatollah ** **Noori ** **Hamedani**	**Ayatollah ** **Makarem ** **Shirazi**	**Ayatollah Fazel ** **Lankarani**	**Ayatollah ** **Alavi Gorgani**	**Ayatollah Safi ** **Golpayegani**	**Ayatollah ** **Mazaheri**	**Ayatollah ** **Seyyed Abbas ** **Modaresi**
**Question**									
Is it permissible to use a dying (nearly dead) patient for medical training in skills such as “endotracheal intubation”, “pericardiocentesis” (a pericardium or external membrane of the heart), or “central venous catheter insertion”? It should be noted that some of these actions affect the patient’s appearance (by puncturing the body, etc.), while others do not change the patient’s appearance.	There is not a problem if the dying patient allows it or has previously given the permission to perform such activities on him/her for medical training purposes; otherwise, it is not permissible. Clearly, a *haram* (forbidden) look and touch should be avoided unless there is a necessity.	It is permissible with the patient’s consent, even if it does not have any benefit for the patient. However, it should not expedite death or cause defects to the patient’s organs.	It is not permissible if the aforementioned training is not a conventional necessity.	It is only permissible in cases of necessity and to the extent of that necessity.	It is forbidden in case of physical harm or disrespect to the dying patient. In the absence of harassment or disrespect, if it causes suffering for the patient due to placing a heavy object on his/her body, it is still *makruh* (undesirable, detestable).	It is not religiously correct and not permissible if performed merely for training (and not therapeutic) purposes, or if there is a noticeable physical damage, or the procedure is painful.	There is no problem if such actions are performed for resuscitation or rescue of a person, but In case of soley training purposes, the medical practice is forbidden to be done and this opens up to liability.	It is permissible if useful or necessary for the patient, but if it is done merely for the purpose of training and entails harm to the patient, it is not allowed, unless the patient gives his/her consent.	It is not permissible, because humans’ lives and bodies are respectable, whether they are Muslims or non- Muslims, dying or already dead, warm, or cold corpses.
2. What is the religious law regarding performing the abovementioned procedures on a dying patient in case the patient and the trainee are of the same or different sexes?	There is not a problem if the dying patient allows or has previously allowed such procedures for medical training purposes; otherwise, it is not permissible. Clearly, a *haram* (forbidden) look and touch should be avoided unless there is a necessity.	It is permissible if they are not of the same sex, but the education is effective in protecting Muslims’ lives or improving their level of knowledge.	It is not permissible if it results in harassment to the dying patient.	It is permissible if the training is necessary for a person of the opposite sex, that is, if without such training the trainee will not acquire the required knowledge to protect patients against a threat or disease.	It is forbidden to perform actions that lead to patients’ harassment, whether they are of the same or opposite sex as the performer of the acts. Moreover, if the trainee’s actions involve looking at or touching a* non-* *mahram* (forbidden) dying patient, then the trainee is a sinner.	In case of patient-trainee sex disconcordance, medical practice is intensely forbidden.	The rule is the same as mentioned in the answer to the first question, even if they are of the same sex.	It is permissible for the same sex and for the opposite sex if it involves no looking and touching, unless there is a necessity.	----
3. Is it necessary to obtain the consent of the patient or that of his/her guardians to perform these procedures? Is this legitimately applicable?	There is not a problem if a dying patient allows or has previously allowed such procedures for medical training purposes; otherwise, it is not permissible. Clearly, a *haram* (forbidden) look and touch should be avoided unless there is a necessity.	Guardians’ consent does not suffice.	-----	Permission is not required in case of necessity; otherwise, there should be consent.	It is forbidden in case of harassment and suffering for the patient. Moreover, the guardians of a dying or nearly dead patient are not allowed to give permission for training activities to be performed on him/her, as they simply cannot make a decision at that moment.	The will or consent of the patient’s guardians does not affect the religious ruling.	The aforementioned procedures must be performed with the patient’s consent, provided that they do not expedite death; however, if the patient is not fully conscious, his/her consent or the consent of the relatives is not beneficial.	It requires permission.	-----
4. Can patients allow the abovementioned procedures to be performed on their nearly dead bodies by writing a will? Will this be a legitimately acceptable or valid will?	There is not a problem if a dying patient allows or has previously allowed such procedures for medical training purposes; otherwise, it is not permissible. Clearly, a *haram* (forbidden) look and touch should be avoided unless there is a necessity.	The patient can make such a will and it is acceptable if the conditions in the answer to the first question are met.	It is permissible if specified in the will and with the consent of the patient’s guardians.	It is permissible in case the patient is informed, his/her consent is obtained, and there is no harm to his/her health or no delay in the process of recovery; otherwise, it is not permissible.	There is not a problem in case the patient has drawn up a will and if there is not a particular *mafsadah * (harm, evil); clearly, it is permissible to act upon the patient’s will.	The will or consent of the patient’s guardians does not affect the religious ruling.	the patient’s will is only acceptable after his/her death.	It is applicable.	-----
5. Are there Islamic conditions or considerations regarding the use of a dying patient for educational purposes? What are those conditions?	There is not a problem if a dying patient allows or has previously allowed such procedures for medical training purposes; otherwise, it is not permissible. Clearly, a *haram* (forbidden) look and touch should be avoided unless there is a necessity.	The answer has been clarified.	It is permissible in cases of necessity and with the consent of the patient’s guardians.	It is permissible in case the patient is informed, his/her consent is obtained, and there is no harm to his/her health or no delay in the process of recovery; otherwise, it is not permissible.	It is not permissible under any conditions if it results in the patient’s harassment and distress. It is acceptable, however, in case there is no suffering or *mafsadah * for the patient.	The patient should not be harassed if the procedure is merely educational.	There are no exceptions regarding this issue.	It is permissible with the patient’s consent and if performed for medical training purposes, or to rescue other people’s lives in order to avoid further threat or harm.	-----
6. What are the answers to the abovementioned questions regarding a Muslim patient’s warm corpse (that is, the body of a person who has just died, while it is still warm)?	There is not a problem if such procedures are aimed at rescuing a respectable individual, making medical discoveries, or obtaining information about a disease that threatens other people’s lives.	There is not a problem provided that following the patient’s death, there is no amputation, bleeding, or disrespect.	It is not permissible if it results in desecration of a believer.	It is not acceptable to cause any deformations in a dying patent’s body unless he/she has consciously consented, or the activities are done for his/her benefit. There is also no difference between a Muslim’s warm or cold corpse in this regard.	-----	The abovementioned activities cannot be performed even for training purposes if the corpse is cold.	It is not permissible and requires *diyah* (compensation or blood money).	The consent of the patient’s guardian as well as that of the religious ruler, and coordination with Legal Medicine Organization is required.	It is imperative that the corpse be a non- Muslim, and this is the rule of God and *Tauzeeh-Ul-* *Masail *(Islamic jurisprudence laws). However, *velaee * (guardianship of the Islamic Jurist) government law is another issue.

Therefore, if the clinical instructor is allowed to practice freely and with no disturbances, the community will benefit from these training activities, and many medical challenges that cannot be addressed by using artificial training alternatives such as manikins, simulators and so on will be resolved.

Although some jurisprudents (Alavi Gorgani and Abbasi Modaresi and Safi Golpayegani) have prohibited the use of a dying person for educational purposes, the practice is permissible based on the rule of necessity, as the majority of the jurisprudents have endorsed it (See Table 1). It should be noted that Alavi Gorgani and Safi Golpayegani consider these activities acceptable on the condition that they are performed for resuscitation or to rescue a patient.

In response to the *istiftas* most jurisprudents (Sistani, Khamenei, Mazaheri, & Nouri Hamedani) stated that in order to perform educational procedures on a dying person, it is necessary to obtain their permission. Moreover, the permission must be given by the person himself, and the consent of relatives is not sufficient (Safi Golpayegani, Sistani & Fazel Lankarani). It can be concluded that based on the principles of *ibaha* and *hilliat* (permissiveness), such procedures are permissible only with the patient’s consent. According to the rule of *la-zarar* (no harm, i.e., there must be no injury or loss in Islam) as well as the necessity of respect for human dignity, prominent authorities have implicitly granted the permission to use a dying patient for training purposes, provided that the procedures do not ensue physical or mental harm. When all aspects are taken into consideration, however, trainers are guaranteed for any harm incurred on patient during training period. Some of the jurisprudents (for instance Safi Golpayegani) have ruled in favor of guaranty in the form of the principle of *la-zarar* for all personal and social interactions of human beings. This ethical principle, which pertains to human behavior in relation to oneself and others, can be extended to the field of medical education as well. Since human wisdom decrees no harm to others, it rules for compensation and the responsibility to redress the loss (2). Although certain circumstances and conditions are required, education on the bedside of a dying patient is acceptable in the light of the principle of expediency, because the benefit of the community is important. 

Based on indications of conscience, necessity and wisdom, the sort of relationship that humans have with their bodies is of ownership and monarchy type, which has also been confirmed by jurist-consults and lawyers (3). In juridical texts, the nature of this relationship is described as ownership (inherent property and credit) and monarchy (authority). There are different views in this regard, for instance some jurisprudents have identified a relationship (ownership) between human beings and their body parts (4), although there is no general consensus on the issue. In addition, Allameh Tabatabai in the interpretation of the first verses of Hamd Surah and the term “*Malik*” (owner), has pointed out that things like body parts and the five senses have no self-dependent existence. In other words, they have no self-dependent existence and do not belong to the person independently, but they are his property. In fact, we are the true owners of our body parts due to existential domination (5). This attention to the human developmental state emphasizes an intrinsic relationship between a person and his/her body parts and consequently ownership of the body. Therefore, ownership of body parts could be regarded as a relationship of domination or a permanent right established between a human being and his/her body members, which has also been validated by the legislator. Moreover, the person might, by virtue of such a right, assume control of his/her body parts so that he/she uses all the benefits and no one can prevent it (3). Some jurisprudents also believe that in certain cases the title of right and property does not apply to a trader in an act of exchange, and prefer the title monarchy instead. The domination of people over their bodies is the same as property ownership, and can be exercised under any circumstances, provided that it is not forbidden by wisdom and the religion (6). In self-ownership, the principle is a strong endorsement of sovereignty of the individual, and it is applicable unless there is a religious law against it (7). In contrast, some other jurisprudents do not believe in a person’s ownership of his/her body due to reasons such as the meditation of the self, in the sense that the human self is not an efficient cause for the body, and only God can control human body affairs. They consider God to be the efficient cause of creation and the true owner of the body and soul of a human being, whom they regard only as a trustee. For this reason, suicide is not permissible and human beings cannot dominate themselves and their organs because there is no justification for this domination (8). Moreover, they consider organ donation as a case of desecration and self-harm (9). Jurisprudents have expressed different opinions in this regard in cases such as the dissection of a deceased Muslim’s body and *diyah *(wergild, blood money, mulct or compensation for manslaughter in Islam), organ transplant from a corpse, and organ donation as presented in Table 2. 

**Table 2:** Religious *fatwas* of prominent Shiite scholars on dissection of corpses and organ donation by the deceased

***Fatwa***	**Dissection of a ** **Muslim’s Dead Body**	**Dissection based on ** **a Person’s Will**	***Diyah*** ** for ** **Dissection**	**Organ Transplant ** **from a Corpse**	**A Will/Certificate of ** **Organ Donation for ** **Postmortem ** **Transplant**
***Ayatollah***					
Javad Tabrizi (10)	If a non-Muslim’s corpse is not available, delay the dissection upon prudence until a non-Muslim’s corpse is found even by payment of any type.	Will is not valid	----------	--------	Will is not valid
Montazeri	It is acceptable if it is established that a Muslim’s life depends on it and there is no other alternative. Otherwise, it is not permissible (11).	It is not unlikely to be permissible and acceptable (11).	*Diyah* is obligatory if dissection is performed for educational purposes and a Muslim’s life does not depend on it (12).	It is permissible in case it may protect a Muslim’s life, but *diyah* is required (12).	----------
Vahid Khorasani (13)	It is not permissible. However, if the life of a Muslim depends upon it and there is no other alternative, dissection is acceptable, but *diyah* is required.	-------	*Diyah* is required.	It is not permissible. If a Muslim’s survival is at stake, the transplant of the body organ is allowed, but *diyah* is required.	The validity of the will is questionable.
Khoei	It is permissible if a Muslim’s survival depends on it and dissection of a non- Muslim’s body or that of a suspicious of being Muslim is not possible and there is no other alternative to keep the Muslim alive (14).	The will could permit both the practice and removal of the *diyah* since they are both related to respect for a Muslim’s body and there is no disrespect in acting upon the will (11).	*Diyah* is required (14), but if there is a will, there is no need for *diyah* (11).	It is permissible if the survival of a Muslim depends on it, but the person who performs the procedure must pay *diyah* (14)	It is allowed and in this case there is no need to pay *diyah* (14).
Imam Khomeini (15)	Dissection is forbidden and not permissible for educational purposes, and if it is not performed to save a Muslim’s life, then *diyah* is required.	-------	If the survival of a Muslim depends on dissection, it is not unlikely that *diyah* is not required; however, *diyah* may be required upon prudence.	It is allowed only to save the life of a Muslim, but *diyah* is required upon prudence.	------
Madani Tabrizi	Dissection of a deceased Muslim’s body is not permitted even for the purpose of acquiring knowledge in medicine (16).	The will is not legitimate and the act is not lawful (11).	*Diyah* is required (16).	It is not permissible and *diyah* is required. If a Muslim’s survival is at stake, however, organ transplant is allowed, but *diyah* is required (16).	The validity of the will is questionable (16).
Mousavi Ardebili (17)	It is acceptable if used for diagnostic purposes and to acquire knowledge in medicine, or to save other people’s lives, provided that it is the only way to achieve the mentioned skills. The consent of the patient or his/her guardian is required if possible.	-------	-------	It is permissible if it will preserve a Muslim’s life and *diyah* is not required.	It is permissible if the maintenance of an important and effective organ of a Muslim depends upon it, especially if the dead patient has allowed it in his/her will.
Fazil Lankarani	If saving a Muslim’s life depends on dissection and a non-Muslim’s corpse is not available, then it is acceptable. However, it is not permissible merely for educational purposes and if the life of a Muslim does not depend on it (18).	It is not unlikely that the will is valid (18). If the patient has made a will to allow an act that entails rational benefits, dissection is acceptable, and execution of will is apparently obligatory (11).	*Diyah *is required upon prudence (11).	It is not permissible to remove the organs of a deceased Muslim unless another Muslim’s life or health depends on it, and a non-Muslim’s corpse is not available, which would be permissible in this case. *Diyah*, however, is required upon prudence (18).	If preserving a Muslim’s life depends on an organ transplant from a Muslim’s corpse and a non-Muslim’s corpse is not available, the transplant is permissible and the will is valid. *Diyah *is required upon prudence under the circumstances (18).
Bahjat (19)	The dissection of a deceased Muslim’s body is permissible under two conditions: 1) If the life of one or more Muslims depends on it. 2) If the dissection of a non-Muslim is not possible. Therefore, if the dissection is merely for educational purposes and no Muslim’s life is at stake, it is not permissible to dissect the Muslim, and *diyah* is required.	The will is not valid.	If the dissection is merely for educational purposes and no Muslim’s life is at stake, it is not permissible and *diyah* is required.	Transplant from a Muslim’s corpse is permissible only if another Muslim’s life depends on it and it is not possible to find a non-Muslim donor. Obtaining permission from the dead person’s guardians is needed upon prudence.	If a person wants to donate an organ such as the kidney, or expresses a wish to do so after his/her death by writing a will, whether in exchange for money or for free, it is permissible if a Muslim’s life depends on receiving that organ and it is not possible to obtain it from a non-Muslim.
Araki (11)	It is not permissible to dissect a Muslim’s corpse.	The will is not valid.	----------	It is not allowed to dissect, cut off, or obtain an organ from a Muslim’s corpse.	---------
Khamenei (20)	There is no problem in dissecting the corpse if it is done to rescue a respectable person, make medical discoveries that are necessary for the community, or obtain information about a disease that threatens people’s lives. However, it is imperative not to use a Muslim’s corpse as far as possible.	It is inconsequential in case of necessity of the dissection.	If possible, a non-Muslim’s body must be used rather than a Muslim’s, and *diyah* is not required if the dissection is absolutely necessary*.*	It is acceptable to use a dead person’s organs for transplant to save another person’s life or to treat his/her disease.	It is permissible to use the organs of the deceased for transplant to save another person’s life or to treat his/her disease. There is no problem if a person wishes to make a will regarding this issue, unless the removal of those organs causes mutilation or disrespect of a corpse by convention.

According to table 2 above, most of the jurisprudents did not allow the dissection of Muslim bodies and ordered *diyah* to be paid. Nevertheless, they mostly declared it permissible where necessary, that is, if a Muslim’s life depended on it and there were no other alternatives. Moreover, they did not consider organ transplant from a corpse permissible and found it acceptable only when performed to preserve the life of a Muslim, and they did not confirm the validity of a will written about the issue. On the other hand, according to table 1, in the light of the rule of authority, the consent of the dying person is applicable in education. In other words, it is advised to respect the patient’s rights and to obtain his/her consent (21), that is, to observe the right of decision-making and free will. 

There is also a conflict between the two views expressed in table 1 (on the educational use of a dying person, which is permissible by consent) and table 2 (on the dissection of a Muslim’s body, which is not permissible unless it is necessary). It can be concluded that because of the social benefits of clinical education as well as the right that a person has on his physical integrity, injury, battery or any physical harassment is forbidden and results in civil and penal liability (22). Moreover, it is inferred from the granting of retaliation, pardon or *diyah* in the case of crimes against the self that human beings have a legal right to their bodies throughout their lifetime (23), and these rights are transferable. Patients’ consent to allow the educational use of their bodies is also acceptable in the light of this right, and therefore training activities are in accordance with this right of patients if they express their consent in their will. 

## Conclusion

An investigation of Islamic jurisprudential rules and the views of prominent contemporary Shiite jurisprudents obtained through *istiftas* regarding medical procedures on dying patients indicate that patients’ rights should in no way be violated. 

Some jurisprudents do not recognize the validity of a dying patient’s will regarding training activities on his/her body. Considering the different viewpoints described above, it can be concluded that on the one hand, medical training is permissible on a nearly dead patient especially if he/she has allowed it by writing a will. It may also be inferred from the opinions of the jurisprudents that using dying patients for teaching medical procedures is contingent on conditions such as necessity and lack of harm. Thus, in order to preserve public interest and to protect the integrity of the dying person, medical training is permissible under certain circumstances. Nevertheless, no specific authority has been specified to identify issues such as necessity, which is a matter of expediency based on time and place requirements, and normally determined by the medical trainer. In addition, lack of a comprehensive and transparent law in this area also causes challenges because juridical provisions may or may not be compatible with the norms of medical ethics. Therefore, it is essential to codify a law in accordance with the following matters: aims of Sharia, personal characteristics and identity, training transcendental students, providing community benefits and Islamic system. It is necessary for medical teachers and staff to be informed on the Islamic issues concerning the use of a dying patient for medical training so they can consider Islamic teachings while providing suitable education for trainees. Although it is the responsibility of specialists in this field to identify instances of harm in medical affairs, a strong relationship between the two areas of medicine and jurisprudence will result in more successful medical education. Therefore, a better interaction between medicine and jurisprudential rules requires that recommendations of the latter be more prominent in medical education. As a result, a dying patient may be used for medical education only when all the following conditions are met:

1. The necessity of performing medical procedures on a dying patient is established;
2. No alternative method is available;
3. The training procedure is carried out to the extent of necessity;
4. The patient’s informed consent or will is obtained in advance to perform the medical procedures;
5. The patient’s dignity is preserved;
6. The procedure is performed under the supervision of a clinical practitioner;
7. Harassment and any form of harm or distress resulting in a prolonged dying process are avoided.
